# Effectiveness and cost-effectiveness of cognitive adaptation training as a nursing intervention in long-term residential patients with severe mental illness: study protocol for a randomized controlled trial

**DOI:** 10.1186/s13063-015-0566-8

**Published:** 2015-02-12

**Authors:** Annemarie PM Stiekema, Piotr J Quee, Marian Dethmers, Edwin R van den Heuvel, Jeroen E Redmeijer, Kees Rietberg, A Dennis Stant, Marte Swart, Jaap van Weeghel, André Aleman, Dawn I Velligan, Robert A Schoevers, Richard Bruggeman, Lisette van der Meer

**Affiliations:** Department of Rehabilitation, Lentis Center for Mental Health, Lentis Psychiatric Institute, Lagerhout E35, 9741 KE Zuidlaren, The Netherlands; Rob Giel Research Center, University of Groningen, University Medical Center Groningen, Hanzeplein 1, 9713 GZ Groningen, The Netherlands; Department of Depression, PsyQ, Lentis Psychiatric Institute, Hereweg 76, 9725 AG Groningen, The Netherlands; Department of Epidemiology, University of Groningen, University Medical Center Groningen, Hanzeplein 1, 9713 GZ Groningen, The Netherlands; Research Department, Lentis Psychiatric Institute, Hereweg 80, 9725 AG Groningen, The Netherlands; Department of TRANZO, Tilburg School of Social and Behavioral Sciences, Dijk en Duin Psychiatric Institute, Tilburg University, Warandelaan 2, 5037 AB Tilburg, The Netherlands; Department of Neuroscience, University of Groningen, University Medical Center Groningen, Antonius Deusinglaan 2, 9713 AW Groningen, The Netherlands; Department of Clinical Psychology and Experimental Psychopathology, University of Groningen, Grote Kruisstraat 2/1, 9712 TS Groningen, The Netherlands; Division of Schizophrenia and Related Disorders, Department of Psychiatry, University of Texas, 7703 Floyd Curl Drive, San Antonio, TX USA; University of Groningen, University Medical Center Groningen, University Center of Psychiatry, Hanzeplein 1, 9713 GZ Groningen, The Netherlands

**Keywords:** Cognitive adaptation training, CAT, Functioning, Severe mental illness, Schizophrenia, Quality of life, Nursing intervention, Cognitive remediation

## Abstract

**Background:**

Despite the well-known importance of cognitive deficits for everyday functioning in patients with severe mental illness (SMI), evidence-based interventions directed at these problems are especially scarce for SMI patients in long-term clinical facilities. Cognitive adaptation Training (CAT) is a compensatory approach that aims at creating new routines in patients’ living environments through the use of environmental supports. Previous studies on CAT showed that CAT is effective in improving everyday functioning in outpatients with schizophrenia. The aim of this study is to evaluate the effect of CAT as a nursing intervention in SMI patients who reside in long-term clinical facilities.

**Methods/Design:**

This is a multicenter cluster randomized controlled trial comparing CAT (intervention group) as a nursing intervention to treatment as usual (control group). The primary goal is to evaluate the effectiveness of CAT on everyday functioning. Secondary outcomes are quality of life, empowerment and apathy. Further, an economic evaluation will be performed. The study has a duration of one year, with four follow-up assessments at 15, 18, 21 and 24 months for the intervention group.

**Discussion:**

There is a need for evidence-based interventions that contribute to the improvement of the functional recovery of long-term residential patients. If our hypotheses are confirmed, it may be recommended to include CAT in the guidelines for SMI care and to implement the method in standardized care.

**Trial registration:**

Nederlands Trial Register (identifier: NTR3308). Date registered: 12 February 2012.

## Background

The term severe mental illness (SMI) defines a range of psychiatric disorders, characterized by serious mental, social and vocational problems that influence each other and create a need for long-term continuous care by mental health professionals [1]. The majority of the SMI population suffers from schizophrenia or related psychotic disorders, which are characterized by positive symptoms (such as hallucinations and/or delusions), negative symptoms (such as amotivation, social withdrawal and/or blunted affect) and cognitive deficits (such as planning, memory and/or attention issues). Furthermore, people with autism, obsessive compulsive disorder, personality disorder, bipolar disorder or major depression are also considered to be suffering from SMI when they follow a severe course of the illness [1]. In the Netherlands, patients with SMI are admitted to long-stay facilities when they are unable to live independently and need more support than can be provided at assisted or sheltered living facilities. Based on the most recent estimations, approximately 10% of the adult SMI population is living in long-stay clinical facilities in the Netherlands [1,2]. The degree of care dependency strongly relates to everyday functioning [3], and is predicted by cognitive impairments, common in the majority of SMI patients [4]. Despite the strong relationship between cognition and everyday functioning, evidence-based interventions are scarce, especially for those who reside in long-stay clinical facilities. Two main approaches can be distinguished with regard to interventions for cognitive problems in SMI: restorative and compensatory.

Restorative approaches, such as cognitive remediation, aim to improve functional outcomes through enhanced cognitive functioning. In cognitive remediation, people are typically trained using computer training software. Cognitive remediation techniques have been extensively studied and though they attain small positive effects on cognition, recent (meta-analytic) studies demonstrated that these improvements do not generalize to functional improvements [5,6]. Significant and larger effect sizes are achieved when cognitive remediation is combined with some form of rehabilitation program, which questions the specific therapeutic effects of cognitive remediation programs on global and real-world functioning [5,6].

Compensatory approaches do not aim to improve cognitive functions per se. Instead, they aim to bypass the cognitive deficits that hinder patients in performing their activities of daily living. Cognitive adaptation training (CAT) is such an approach [7,8]. CAT is an in-home intervention that targets real-world problems in a real-world setting, namely the patient’s own living environment. With the use of environmental supports, the patient is taught new routines that appeal less to cognitive functions. Furthermore, CAT interventions are strongly individualized, taking into account the specific problems in activities of daily living of the individual patient. Effects of CAT include improvements in everyday functioning, quality of life, medication adherence and relapse in outpatients with schizophrenia [7-9].

CAT starts with composing an individually tailored treatment plan, based on the patient’s specific problems with respect to behavior, cognitive or executive functioning and everyday functioning. If the patient’s behavior type is predominantly characterized by apathy (amotivation, lack of initiative and/or lack of goal-directed behavior), compensation strategies will focus on cueing and prompting behavior. Patients whose behavior is predominantly characterized by disinhibition (easily distracted by irrelevant stimuli and/or behaving inappropriate in a situation) may benefit from removal of distracting stimuli. For patients with a mixed behavior type, both intervention strategies can be used. The form of the intervention is based on the level of executive functioning of the patient (such as planning, goal-directed behavior and mental flexibility). Interventions for patients with poor executive functioning are more easily noticeable (for example, larger signs), more specific (for example, stepwise instructions) and more numerous, compared to interventions for patients with fair executive functioning, which can be more subtle (for example, smaller signs) and less specific (for example, global instructions).

Information about the patient’s cognitive strengths and weaknesses in attention and memory is used to further individualize the interventions. For example, if a patient has strong visual attention but their auditory attention is weak, they would probably benefit more from visual cues and/or signs than from verbal reminders. The specific individual problems in everyday functioning determine the individual goals at which the interventions are directed. Commonly used environmental supports are watches, calendars, electronic devices, signs, household supplies (such as boxes and cleaning supplies) and means of transportation (for example, the bus or a taxi pass). A CAT manual includes examples of specific environmental supports for 16 domains of daily living [10], and was slightly adapted (by authors APMS and PQ) for practical use by psychiatric nurses.

Despite the well-established effectiveness of CAT for outpatients, it remains unclear whether long-term residential patients can benefit from CAT in a similar way. In this population, the cognitive and functional deficits can be considered to be most severe. Therefore, CAT may bridge the gap between the current knowledge about these deficits and the lack of evidence-based interventions for this patient group. At the same time, long-stay facilities may be better able to deal with the need for ongoing support, which is necessary to maintain the functional improvements made with CAT [9,11].

In the usual care of long-stay facilities, ongoing support is often provided by psychiatric nurses. Supporting patients in performing everyday tasks is part of their daily working routine. While in previous studies CAT was delivered by psychologists with a minimum of a bachelor’s level education, the current study aims to train psychiatric nurses for CAT. Involving nurses should integrate CAT in the daily working routine and thereby provide the continuous support which is necessary to sustain improvements that are made with CAT. A pilot study conducted by our group provided tentative evidence that CAT as a nursing intervention can improve everyday functioning in long-term residential patients in the Netherlands [12]. Furthermore, a recent Canadian study demonstrated that case manager support can sustain CAT interventions after a period of CAT-specialized intervention [13].

The abovementioned studies only included patients with a diagnosis in the schizophrenia spectrum, which leaves the effect of CAT on SMI patients with a different diagnosis unknown. However, cognitive impairments are also present in patients with other psychiatric disorders [14]. Therefore we expect that SMI patients with cognitive deficits, but without the psychotic symptoms that characterize schizophrenia, may similarly benefit from CAT.

The primary goal of the current study is to assess the effect of CAT as a nursing intervention on everyday functioning in long-term residential patients with SMI. The secondary goals are to evaluate the effect of CAT on quality of life, empowerment and apathy. Follow-up measurements will be conducted to evaluate whether obtained effects on primary and secondary outcome measures can be retained, as well as to evaluate the feasibility of integrating CAT support into the daily working routine of the nurses. We expect CAT as a nursing intervention to lead to improvements in everyday functioning and apathy, and thereby an improved quality of life for patients with SMI. As CAT is not designed to alter cognitive functioning (rather to bypass cognitive problems) or to improve positive symptoms, we do not expect these to change. Finally, we will assess the long-term cost-effectiveness and cost-utility of the intervention. Importantly, in a time where available funding for mental healthcare is diminishing, evaluation of the costs and benefits of CAT is necessary. With this information available, mental health institutions will be able to make well-considered decisions with regard to the type of treatments and interventions that they offer.

## Methods/Design

The methods and design of the intervention study are described using the Consolidated Standards for Reporting Trials (CONSORT) statement and the extension for reporting cluster randomized controlled trials [15].

### Design

This is a two-arm, multicenter, cluster randomized controlled trial assessing CAT as a nursing intervention in long-term residential patients with SMI. Patients in the intervention group will receive CAT in addition to treatment as usual ((TAU) CAT + TAU) and will be compared to the control group who will only receive TAU. Assessments take place at baseline and every three months thereafter. This study has a duration of one year. For the intervention group, four additional assessments take place at 15, 18, 21 and 24 months in order to investigate the long-term effects.

The Medical Ethics Review Committee of the University Medical Center Groningen in the Netherlands approved the study design, research protocol, information brochure and informed consent procedure (approval number M13.143271). The study is funded by a NutsOhra grant (grant number: 1303–041).

### Recruitment and inclusion of patients

A total of 100 long-term residential patients suffering from SMI will be included in the study. Patients who participated in the pilot study or patients who are under 18 years of age are excluded from participation. There are no other criteria held.

Patients are recruited from the long-stay departments of three institutions in the Netherlands: Lentis Psychiatric Institute (Zuidlaren), Dijk en Duin Psychiatric Institute (Castricum) and GGZ Drenthe (Assen). The departments typically provide long-term pharmacological and psychosocial treatment and focus on rehabilitation and recovery for adult SMI patients. Admitted patients have often insufficiently recovered during several previous hospitalizations and/or periods of sheltered living, and experienced educational, vocational and social problems early in life. The majority of the patients suffer from psychotic disorders. Other diagnoses include severe major depression, personality disorders, bipolar disorder and autism.

Patients are informed about the study by nurses from the concerning department. Patients are informed that participation is voluntary and that they are free to withdraw at any time without providing a reason and without consequence for the usual treatment. Questions can be directed at the nurses or the researchers (contact information is provided in a patient folder). Participants are required to provide written informed consent at baseline.

### Sample size calculation

This sample size is based on a power analysis using the effect size (0.79) calculated from Multnomah Community Ability Scale (MCAS) scores from our pilot study [12]. To detect a significant change in daily functioning measured by the MCAS with α set at 0.05 and a power of 0.9, 35 patients should complete the intervention. Studies have shown that withdrawal from psychosocial interventions in psychiatric patients can be substantial. Therefore we have taken into account a conservative drop-out rate of 30% and will include at least 50 patients in each condition. Analyses are performed when both groups completed the assessments at 12 months and at the study end when follow-up assessments of the intervention group have been completed (24 months).

### Randomization

With regard to the randomization procedure, several issues were to be considered. Each location employed several nursing teams, with separate offices, patient units and caseloads. Cluster randomization was applied on a nursing team level for each of the three institutions. This resulted in an equal distribution in the number of nursing teams allocated to CAT and TAU within each institution. Not only does this approach prevent contamination between nurses in the intervention and control groups, it also enhances implementation of the intervention since all nurses within each intervention team are trained in CAT and regular nursing meetings can be used for informing colleagues about the progression of the intervention. The nursing teams are randomly allocated to either the intervention or the control group by an independent staff member (without interest in or knowledge about the outcome of the allocation) who blindly draws a ticket from a basket containing a CAT ticket and a blank ticket. No inclusion or exclusion criteria are applied for nurses.

### Cognitive adaption training nurses and specialists

Nurses in the intervention teams are responsible for setting up and evaluating the intervention for a maximum of one to three patients, while all nurses are responsible for supporting the daily use of interventions of all patients. Hereafter, the term CAT case manager refers to the former role, while the term nurse refers to the general tasks of all nurses in the team. CAT specialists are professionals educated to at least bachelor level, who have knowledge about the relationship between cognition and general functioning, and who are knowledgeable with regard to the underlying principles of CAT. Besides supervising CAT case managers in the development and implementation of the intervention for each individual patient, CAT specialists guard the underlying principles of CAT in the interventions that are applied. CAT specialists APMS and JER, both psychologists at Lentis Psychiatric Institute, were trained by Dr Velligan, the developer of CAT. They provided additional training for CAT specialists at the other institutions.

### Blinding

Blinding will take place at the level of assessors of outcome variables. Blinding of patients and nurses is not possible due to the nature of the intervention. Similarly, blinding of the researcher is not possible due to the researcher’s (APMS) role in the training of nurses and CAT meetings with nurses. Research assistants (psychology students educated to at least bachelors level) are trained to administer the assessments. They are blind to the treatment arm in which the patient is included and do not receive any information with regard to the content and aims of the intervention. Nurses and patients are requested not to disclose any information about the allocation of conditions or the content of the intervention to the students. Students are instructed to inform the researcher immediately when they suspect blinds are broken, in which case the measurement is finished by another student who is still blind to the allocation of the patient. Furthermore, guessing of the allocation group by raters after each assessment will be used to evaluate whether blinding was successful.

### Tests and measures

#### Testing procedure

All participants (intervention and control group) are measured at baseline and after three, six, nine and 12 months. Additionally, to determine whether any obtained results in the intervention group can be retained, follow-up assessments are planned after 15, 18, 21 and 24 months. Although CAT is not designed to alter cognitive functioning (rather to bypass cognitive problems) nor to improve symptoms, cognitive functioning and symptomatology are measured again at six and 12 months to evaluate any unexpected changes. The schedule of enrollment, interventions and content of the assessments is shown in Table 1. For all patients, interviews and cognitive tests are assessed in separate sessions. In the intervention group, environmental and functional problems in everyday functioning are assessed in an additional session. Level of everyday functioning is chosen as the primary outcome. All tests and measures are described below. Secondary outcome measures are quality of life, empowerment and apathy. Assessment outcomes for the control group will not be used in their treatment plan for the duration of the trial.

**Table 1:** **Schedule of enrollment, interventions and assessments**

	**Study period**									
		**Intervention period**					**Follow-up** ^**b**^			
**Time point** ^**a**^	**< T**_**0**_	**T**_**0**_	**T**_**3**_	**T**_**6**_	**T**_**9**_	**T**_**12**_	**T**_**15**_	**T**_**18**_	**T**_**21**_	**T**_**24**_
**Enrollment:**										
Randomization	X									
Informed consent		X								
**Groups:**										
Control (TAU)		X	X	X	X	X				
Intervention (TAU + CAT)^c^		X	X	X	X	X	X	X	X	X
**Assessments:**										
Environmental/functional problems^b^ and behavior type		X								
Clinical and cognitive characteristics		X		X		X				
Demographical information		X								
Everyday functioning		X	X	X	X	X	X	X	X	X
Quality of life and empowerment		X		X		X				
Apathy		X		X		X				
Healthcare costs		X	X	X	X	X	X	X	X	X

^a^Number illustrates number of months after baseline.

^b^Assessed for the intervention group only.

^c^ *Abbreviations*: *TAU* treatment as usual, *CAT* Cognitive Adaptation Training.

#### Demographical information

Demographical information (date of birth, gender, nationality, level of education, main diagnosis, comorbid diagnoses, age of onset, psychiatric disorders of parents and siblings, relevant medical events, neurological disorders and alcohol and drug use) is partly obtained from the patient file and completed at baseline assessment (T0).

#### Severity of current psychotic symptoms

The presence and severity of psychotic symptoms is measured using the Positive and Negative Syndrome Scale (PANSS) [16] by trained blind raters (psychology students educated to at least bachelors level). The PANSS is a semi-structured interview that consists of three subscales: positive symptoms, negative symptoms and general psychopathology. The PANSS is a widely used instrument with good psychometric properties [16]. The PANSS is administered for patients with a psychotic disorder or a disorder with psychotic features only.

#### Behavior type

The Frontal Systems Behavior Scale (FrSBe) [17], which is filled out by the CAT case manager, is used to determine the behavior type. This scale consists of 46 statements on apathy, disinhibition and executive functioning. Each statement can be scored on a scale ranging from one (almost never) to five (almost always). The apathy and disinhibition scores determine whether the patient’s behavior type is best characterized as apathy, disinhibition or mixed (combination of apathy and disinhibition).

#### Executive functioning

The level of executive functioning is determined with the Modified Card Sorting Test (MCST) [18] and the letter fluency task [19]. The perseveration and categories score of the MCST and the total score of the letter fluency task are used to determine the level of executive functioning. A division is made between poor (problems with planning and performing the steps of basic everyday tasks such as showering and dressing) and fair (problems on higher level functions, such as management of money and work skills). When the participant is not able or willing to participate in the tests, the score on the executive subscale of the FrSBe is used to determine the level of executive functioning.

#### Cognitive strengths and weaknesses

Complementary cognitive tests are administered to determine strengths and weaknesses in visual attention (Picture Completion; Wechsler Adult Intelligence Scale IV (WAIS-IV)) [20], auditory attention (Digit Span forward; WAIS-IV) [20], working memory (Digit Span Backward; WAIS-IV) [20] and verbal short term memory (Word Learning Task) [21]. Strengths and weaknesses in auditory attention and working memory are determined with cut-off scores identical to those used by Dr Velligan (personal communication, A.P.M. Stiekema) (auditory attention: <4 = weak, >7 = strong and 4 to 7 = average; working memory: <2 = weak, >5 = strong and 2 to 5 = average). For visual attention, we use transformed raw scores to scale scores on the picture completion task according to the Dutch norms [20] and set cut-off points for the scale scores (<5 = weak, >10 = strong and 5 to 10 = average). Finally, for verbal short term memory, we use the available Dutch norms [21] to set cut-off points at percentiles (<25 = weak, >50 = strong and 25 to 50 = average).

#### Environmental and functional assessment

The individual problems with everyday functioning are assessed with the Environmental and Functional Assessment (EFA) by Velligan *et al*. [10]. The EFA is an extensive interview on daily activities, functional skills and the presence of necessary skills to perform these activities. We slightly adjusted the EFA so that the given examples reflect situations that are common for the study population in the Netherlands. In addition, we now explicitly ask for the patient’s wishes with regard to the every tasks. The EFA is administered in the patient’s residence by a CAT specialist together with the CAT case manager. If necessary, the CAT case manager supports the patient in answering the questions during the interview.

#### Everyday functioning

Level of everyday functioning is measured with a number of instruments. First, the Multnomah Community Ability Scale (MCAS) [22] is used to enable us to compare results with previous CAT studies. The MCAS is a semi-structured interview containing 17 items and assesses a variety of domains of community adjustment. As reports of mental healthcare professionals who are in frequent contact with patients tend to be more linked to performance on everyday tasks than self-reports [23], a comparison of patients’ reports and nurses’ observations (measured with observational questionnaires described below) is used to determine possible discrepancies between answers and to adapt MCAS scores if necessary. The instrument was translated into Dutch (PQ) and back-translated by a professional translator. The English version has proven to be reliable and valid [24].

The second instrument used to measure everyday functioning is the Social and Occupational Functioning Scale (SOFAS) [25]. The SOFAS is a reliable and valid instrument for measuring social, occupational and interpersonal functioning on a single-item scale (range: 0 to 100) [26].

Third, engagement in work-related activities is used as an indication of everyday functioning. Engagement in work-related activities is measured in number of partial days a week (a partial day consists of three hours) and is registered daily by the staff.

Finally, two observational questionnaires are filled out by a nurse (the CAT case manager in the intervention group; for the intervention group individual nurses are allocated between one and three patients for whom to complete the observational questionnaires). The Social Functioning Scale-other (SFS) [27] measures functioning in society. It contains seven subscales: (1) social engagement, (2) interpersonal behavior, (3) pro-social activities, (4) recreation, (5) independence-competence, (6) independence-performance and (7) employment and/or occupation. The SFS has good psychometric properties [27]. The second observational questionnaire is the Life Skills Profile (LSP) [28]. The LSP is a questionnaire consisting of 39 questions on a four-point scale (lower scores indicate better life skills) and measures a range of aspects related to successful community or hospital living: (1) self-care, (2) non-turbulence, (3) social contact, (4) communication and (5) responsibility. The scale has shown good psychometric properties when completed by case workers and residential staff [29].

#### Quality of life

Quality of life is measured with the Short Form Health Survey (SF-12) [30]. The SF-12 is a self-report questionnaire with 12 items measuring subjective physical, psychological and social well-being. The SF-12 has good psychometric properties for use in SMI populations [31].

#### Empowerment

Empowerment is measured by a self-report questionnaire, the Dutch Empowerment Questionnaire ((DEQ) *Nederlandse Empowerment vragenLijst*) [32]. Three of the six subscales are used in the current study, namely professional help, self-knowledge and belonging. Items are scored on a five-point scale ranging from one (strongly disagree) to five (strongly agree). The validity and reliability of the DEQ is sufficient [32].

#### Apathy

The avolition-apathy subscale of the Scale for the Assessment of Negative Symptoms (SANS) [33] is administered to measure apathy. The four items of this subscale can be rated from 0 (absent) to four (severe). The SANS is a valid and reliable instrument [34].

Motivation to engage in activities is assessed using the motivation subscale from the Negative Symptom Assessment, a semi-structured interview (NSA-16) [35]. The motivation subscale contains four items that can be rated from one (no evidence of this symptom) to six (severe). The validity and reliability of the NSA-16 is good [36].

### Treatment as usual

Although the institutions vary somewhat in the treatments they offer, all patients have access to pharmacotherapy. The range of psychological, psychosocial and non-verbal therapies (such as psycho-education, cognitive behavioral therapy, Liberman training, peer support groups, psychomotor therapy, music therapy, creative arts therapy and sports groups), as well as educational or work projects (such as a framing center, graphic design and copy center, a farmhouse, catering and site and garden maintenance) differs somewhat between locations, which will be accounted for in the analyses. For each patient, treatment is aimed at a combination of therapies and daily activities that best matches the needs, goals and wishes of the patient in order to reach an optimal recovery process. Treatment and progress are evaluated at least once per year.

### Cognitive adaptation training: the intervention

#### Preparation: training of nurses and cognitive adaptation training specialists

To optimize the implementation of CAT and to ensure CAT would be included in the working routine, all nurses in the intervention groups were trained in CAT, though not screened for eligibility. Nurses participate in a didactical group training prior to the recruitment of patients. The training is delivered by the CAT specialists who were trained in CAT by Dr Velligan, the developer of CAT (APMS and JER). Translated PowerPoint presentations and video demonstrations of Dr Velligan serve as the basis of the didactical training, to ensure fidelity to the CAT treatment model. The didactical training includes the theoretical background and principles, assessments, use of the CAT manual, intervention strategies, CAT visits and continuity of the intervention. APMS and JER provided an additional training on assessing the EFA, composing the CAT treatment plan (see below) and continuity of CAT in daily care for the CAT specialists at all intervention locations.

#### Designing the cognitive adaptation training treatment plan

The protocol developed by Dr Velligan and her group (personal communication, A.P.M Stiekema, P.J. Quee) is followed to design the CAT treatment plan, with some minor modifications due to the availability of tests translated into Dutch. The CAT plan and starting point of the intervention is based on: (1) behavior type, (2) level of executive functioning, (3) goals for improving everyday functioning and (4) cognitive strengths and weaknesses that are assessed at T0. Upon completion of this assessment, a CAT specialist summarizes the outcomes of the cognitive assessment and discusses these with the CAT case manager during the first CAT meeting. Based on the wishes and problems reported by the patient during the EFA, the CAT specialist and the CAT case manager discuss the priority of problems and make an initial priority of goals that may be targeted with the intervention. In the next step of the intervention process, the CAT case manager discusses the assessments with the patient during the first CAT visit. The patient is encouraged to formulate their own goal, however if they are unable to do so, the CAT case manager suggests a goal according to the priority of the initial CAT plan. If necessary, the priority on the CAT plan is adapted following the wishes of the patient after the first or subsequent CAT visits.

#### Implementing the cognitive adaptation training treatment plan

Once the CAT treatment plan is formed and discussed with the patient, the CAT specialist and CAT case manager discuss possible interventions using the CAT manual and taking individual characteristics into account. Possible intervention strategies are discussed and set up together with the patient when in any way possible. Intervention strategies are limited to one goal at a time to ensure patients are not faced with more than they can handle. After the first intervention the CAT case manager is responsible for setting up and organizing environmental supports and evaluating the interventions with the patients in their caseload. CAT visits to the patients are planned within the daily routine of the nurses. Based on our pilot study, a duration of CAT visits of approximately 45 minutes a week per patient in total (time may be divided over a number of CAT visits) is deemed to be sufficient. In addition, the CAT case manager makes a record of the CAT visits in the patient file, based upon four questions: what interventions worked, what interventions did not work, what changes are made in the intervention strategies and what issues need to be kept in mind.

During the implementation phase (one to two months), the CAT specialist and CAT case manager plan CAT discussion meetings at least once a month. The nursing team as a whole is informed of, and responsible for, and supports the daily use of the interventions of all patients (for example, checking and reporting on whether a recorded message leads to the targeted behavior, or changing a weekly checklist). In the securing phase (up to 12 months), CAT group meetings are incorporated into regular staff meetings for at least another six months, to stimulate the exchange of knowledge and interventions between nurses. Finally, after the securing phase (after 12 months), a CAT focus group including the researchers and clinicians (APM, JER and LM) and a delegation of nurses, will determine the form and frequency of CAT meetings in the follow-up phase (12 to 48 months).

### Statistical analysis plan

#### Primary and secondary outcomes

Mean differences in the outcomes between the intervention and control group will be investigated by applying a marginal linear mixed model (modeling mean scores for both groups) using restricted maximum likelihood estimation to the outcomes at each time point after baseline. The analyses are corrected for baseline scores to eliminate the effect of possible (average) differences between the intervention and control group at baseline. The treatment effect is essentially estimated for each time point by subtracting the estimated mean scores of the control group from the estimated mean scores of the intervention group, reflecting the estimated mean difference between the groups over time.

Sub-analyses will be performed to evaluate individual improvement in everyday functioning. To this end, we will specifically evaluate only those items of the MCAS, SFS and LSP that measure the domain(s) that are recorded as goals in the CAT treatment plan. That is, we will evaluate whether patients have indeed improved on the activities they have been working on with CAT, and for which individually tailored interventions were set up. Sum scores of items of the LSP, and SFS reflecting a certain goal will be computed. Differences in these sum scores over time will reflect whether patients’ performance on these domains have improved, did not change or have deteriorated. Generalized linear models will be used to determine whether there is a statistically significant proportion of improvement target behaviors compared to no change or deterioration.

#### Economic evaluation

An economic evaluation will be conducted alongside the clinical study to assess the balance between costs and health outcomes of CAT + TAU compared to TAU.

#### Healthcare consumption and costs

This study is conducted from a healthcare perspective, since costs within the healthcare system are most relevant for the current study population. Medical costs that will be assessed include costs of the interventions (costs of personnel, materials and housing), inpatient care and medication use. In order to calculate costs, Dutch standard prices for each cost unit [37] are combined with information on healthcare consumption. Healthcare consumption will be prospectively registered for all the included patients by means of a detailed questionnaire adapted to the context of the current study. This questionnaire is completed by each CAT case manager at baseline and at three, six, nine and 12 months for both groups and at 15, 18, 21 and 24 months for the intervention group.

#### Cost-effectiveness and cost-utility analysis

The primary outcome measure of the planned cost-effectiveness analysis (CEA) is the MCAS. Results of the CEA are expressed in terms of incremental costs per point change in MCAS score. Furthermore, a cost-utility analysis will be conducted with quality adjusted life years (QALY) as the primary outcome measure. In order to estimate QALYs, utility scores are derived from the SF-12 [38]. The uncertainty surrounding the cost-effectiveness and cost-utility ratios will be estimated by bootstrap analyses. In addition, cost-effectiveness acceptability curves will be used to inform decision-makers on the probability that CAT is cost-effective.

## Discussion

In SMI care, especially in the chronic stage of the disease, there is a necessity for practical hands-on interventions that target the functional impairments resulting from cognitive deficits in such a way that functional improvements are made, and can also be sustained after a study period. Primarily the latter aspect made us choose an intervention that can be applied by those healthcare professionals who support patients in their daily routine, namely psychiatric nurses. The current, multicenter, cluster randomized controlled trial meets with these needs by assessing the efficacy of CAT as a nursing intervention on everyday functioning in residential patients with SMI. We hypothesize that CAT as a nursing intervention leads to improvements in everyday functioning (primary outcome) and apathy, and thereby in quality of life and empowerment (secondary outcomes).

A strength of this study is that patients are recruited from multiple psychiatric institutions spread across the Netherlands, increasing representativeness and thus increasing generalizability. An additional strength is that it addresses the long-term effects of the intervention (up to two years). The length of the study takes into account that rehabilitation in long-term residential patients is a slow process [39]. Furthermore, by following up on everyday functioning for up to two years (one year after completion of the study), we will evaluate whether CAT has been successfully incorporated in the working routine of nurses. This is important because the nurses will have to provide ongoing support to sustain improvements. Another strength is that the outcomes of everyday functioning are not solely based on self-reports, but are also assessed by the number of work-related activities per week, and by observational questionnaires that are filled out by the CAT case manager. As such, we use various measures to substantiate our impression of the level of everyday functioning of the patients. Furthermore, this approach allows us to collect data even if the patient misses an assessment. Finally, because of our choice not to hold common eligibility criteria, for example with regard to diagnosis or (prior) comorbid psychiatric or somatic disorders, our results will be better generalizable to the whole SMI population, instead of only pertaining to schizophrenia patients. This is important since it increases the clinical importance and external validity of our findings.

Possible weaknesses of the study are the lack of measurements focusing on the nurses who carry out the intervention. Motivation, self-efficacy and empowerment of the nurses may mediate the effect of the intervention, but an absence of appropriate instruments in this regard leaves us unable to take these aspects into account. Moreover, there are few instruments that are specifically designed for measuring everyday functioning of patients residing in long-stay clinical facilities, whose problems and needs may differ from patients living in the community. Finding a suitable instrument is further complicated by the fact that areas for interventions are individually determined and therefore differ for each patient. Due to the global nature of the measures of everyday functioning, our instruments may not be sensitive enough to assess improvements because CAT aims to improve one or two areas of functioning at a time. For this reason, we included the additional sub-analyses in the statistical analysis plan that specifically evaluate improvements in targeted domains (as described in the statistical analysis section).

If evidence yields support for CAT, this may fulfill a need for a hands-on intervention that can contribute to the functional recovery of patients living in long-stay clinical facilities.

## Trial status

Recruitment is currently ongoing. Patient enrollment started in September 2013 and will continue until 100 patients are included in the study.

## Abbreviations

CAT: Cognitive adaptation training
CONSORT: Consolidated standards of reporting trials
DEQ: Dutch empowerment questionnaire
EFA: Environmental and functional assessment
FrSBe: Frontal systems behavior scale
LSP: Life skills profile
MCAS: Multnomah community ability scale
MCST: Modified card sorting test
NSA: Negative symptom assessment
PANSS: The positive and negative syndrome scale
QALY: Quality adjusted life years
SANS: Scale for the assessment of negative symptoms
SF-12: Short form health survey
SFS: Social functioning scale
SMI: Severe mental illness
SOFAS: Social and occupational functioning scale
TAU: Treatment as usual
WAIS-IV: Wechsler adult intelligence scale IV
